# Treating Early-Stage DLBCL on the FLYER: What Lesson for Radiation Therapy?

**DOI:** 10.3389/fonc.2021.686223

**Published:** 2021-06-04

**Authors:** Kelsey Sokol, Amanda McBride, Adam Finn Binder, Pierluigi Porcu

**Affiliations:** Department of Medical Oncology, Division of Hematologic Malignancies and Hematopoietic Stem Cell Transplantation, Sidney Kimmel Cancer Center at Jefferson Health, Thomas Jefferson University, Philadelphia, PA, United States

**Keywords:** DLBCL, favorable, early-stage, lymphoma, CHOP (R-CHOP), radiation

## Introduction

The combination of cyclophosphamide, doxorubicin, vincristine, and prednisone (CHOP) and the anti-CD20 monoclonal antibody rituximab has been standard of care for diffuse large B-cell lymphoma (DLBCL) since 2006, based on results from three randomized Phase III clinical trials: GELA/LNH 98-5, Intergroup/E4494, and MInT (1–3). These trials showed that the addition of rituximab to 6-8 cycles of anthracycline-based chemotherapy increased overall survival (OS) and progression free (PFS) or event free survival (EFS) in both older patients (>60) with advanced-stage DLBCL (GELA/LNH-98-5 and E4494), and younger patients (≤60) with favorable-risk disease, defined as stage II-IV with 0-1 International Prognostic Index (IPI) risk factors or bulky stage I disease (MInT).

The MInT trial showed that 6 cycles of R-CHOP every 21 days (R-CHOP-21) are sufficient in younger DLBCL patients with favorable features and that patients with early-stage DLBCL (Ann Arbor I and II) and no tumor bulk (variably defined) had excellent outcomes without radiation therapy (RT), making R-CHOP x 6 an alternative to R-CHOP x 3 + RT (3). A subset of patients (N=101) with age-adjusted IPI 0 and disease bulk <7.5 cm had very favorable outcomes (PFS and OS at 6 years 89.6% and 94.9%, respectively). Did this population actually need 6 cycles of R-CHOP? Or could fewer cycles be sufficient (without RT)? While the FLYER trial was not designed to directly answer the RT question, it is relevant for RT decisions in clinical practice.

The FLYER trial was an international, randomized Phase III study conducted between December 2005 and October 2016 in the very favorable subset identified in the MInT trial, i.e. “young” patients (age 18-60) with stage I/II (based on CT or PET/CT) untreated CD20-positive aggressive B-cell lymphoma, without bulky disease, and with no age-adjusted IPI risk factors (4). The study enrolled 592 patients, of which 85% had DLBCL. Patients were randomized to receive R-CHOP every 21 days for 4 cycles, followed by an additional two doses of rituximab (n=297), or six cycles of R-CHOP every 21 days (n=295). Radiation was planned only in patients with testicular involvement, as prophylaxis to the contralateral testis.

The study asked whether a reduction in chemotherapy to 4 cycles would be non-inferior to standard treatment with 6 cycles, with a non-inferiority margin of -5.5%. After median follow-up of 66 months, the study met its primary endpoint, with an absolute difference in 3-yr PFS of 3% (96% with 4 cycles R-CHOP plus two doses of rituximab vs 94% with 6 cycles R-CHOP) and no difference in 3-yr EFS or OS (89% vs 89% and 99% vs 98%, respectively). Forty patients progressed or relapsed. Rates of relapse for patients with complete response (CR) or unconfirmed complete response (CRu), based on the 1999 consensus criteria (5), were similar, with no CNS relapses. About two thirds of relapses occurred after 2 years. Fewer adverse events occurred in the reduced chemotherapy group. The rate of secondary neoplasm was similar. Does the FLYER study give us justification to use 4 cycles of R-CHOP, as opposed to 6 (as in MInT) for these patients? An important question that the FLYER study did not directly address is: can we treat some patients with early-stage DLBCL with chemotherapy only, omitting radiation?

## Point

The FLYER trial presents an improved treatment approach for younger patients with early-stage favorable risk DLBCL, decreasing toxicity through radiation omission and abbreviation of chemotherapy.

Prior to this trial, the addition of involved field RT to chemotherapy, or combined-modality therapy (CMT), was employed to allow for abbreviated chemotherapy in early-stage DLBCL. The phase III SWOG 8736 trial initially examined CMT in early-stage disease (6). Patients were randomized to 3 cycles CHOP followed by RT (40-55 Gy to all visible sites of disease) versus 8 cycles CHOP alone. Significant improvement in 4-year PFS (77% vs 64%) and OS (82% vs 72%) in favor of CMT was shown. This led to the widespread adoption of CMT for patients with early-stage DLBCL. The subsequent SWOG 0014 trial, a single arm phase II trial, confirmed excellent outcomes with CMT for early-stage DLBCL in the rituximab era (7).

Others evaluated radiation omission in early-stage DLBCL. The GELA LNH-93-4 trial examined abbreviated chemotherapy without RT in the pre-rituximab era. Patients with early-stage DLBCL and no adverse IPI risk factors were randomized to 4 cycles CHOP followed by RT versus 4 cycles CHOP alone (1). There was no difference in 5-year EFS or OS between the two groups. Notably, patients were >60 years old, a population excluded from FLYER. In the rituximab era, LYSA/GOELAM 02-03 randomized patients age 18-75 years with stage I/II non-bulky (<7 cm) disease to receive RT versus no RT if interim PET/CT (iPET) following 4 cycles R-CHOP showed CR (88% of patients) (8). Patients received a total of 4 (n=187) or 6 cycles (n=148) based on the presence of IPI risk factors. Notably, R-CHOP was given every 14 days. For patients with CR on iPET, 5-year EFS and OS were similar between patients who received CMT versus chemoimmunotherapy alone. Collectively, these trials support RT omission and chemotherapy abbreviation early-stage DLBCL.

Recently, the S1001 trial evaluated a response-adaptive strategy in patients with early-stage DLBCL (9). In this phase II study, 132 eligible patients with early-stage, nonbulky (<10 cm) disease received 3 cycles R-CHOP followed by iPET. If iPET negative, patients received 1 additional cycle R-CHOP for 4 cycles total. If iPET positive, they received RT followed by ibritumomab tiuxetan, an anti-CD20 radioimmunoconjugate. In 128 iPET-evaluable patients, the 5-year PFS and OS were excellent in both iPET-pos (N=14) and iPET-neg (N=114) patients at 86% vs. 89% and 85% vs. 91%, respectively.

Molecular and biologic risk factors were not evaluated in the FLYER trial. However, while cell of origin (COO) and double expressor (DE)/double hit (DH) status are important prognostic factors in advanced-stage DLBCL, the prognostic implication in early-stage is unclear. Three retrospective studies provide some data to address this question. One international study evaluated 192 patients with early-stage DLBCL treated with R-CHOP-like regimens with or without RT (10). Of these patients, 60% were classified as GCB and 40% as non-GCB. The total cohort had excellent outcomes with 4-year PFS 85% and 4-year OS 88%, and COO status did not influence outcomes. This study also evaluated outcomes related to DE/DH status. Although limited numbers, DE (32 patients) and DH (6 patients) status was not associated with inferior PFS or OS. An additional retrospective analysis evaluated the impact of COO on outcomes in 87 patients with early-stage DLBCL treated with R-CHOP 3-4 cycles plus RT (11). Most patients (75%) had GCB phenotype, and IPI was similar between GCB and non-GCB groups. Again, no significant difference in PFS or OS between the two phenotypes was found. More recently, a US multicenter retrospective study evaluated outcomes in 104 patients with MYC-rearranged early-stage DLBCL (including 40 patients with DH) treated with standard R-CHOP vs more intensive immunochemotherapy (IIC = R-DA-EPOCH, R-HyperCVAD/MA, or R-CODOX-M/IVAC) +/- RT. The 2-year PFS and OS were 78% and 86% for the entire cohort, and 74% and 81% for the DH patients, respectively. PFS and OS were similar across treatment groups in the entire cohort and in patients with DH, noting that the addition of consolidative RT yielded a higher CR rate. Furthermore, the ORR in DH patients was comparable between regimens (12). These studies suggest that COO and DE/DH status may not have significant prognostic implications in early-stage DLBCL. Therefore, the lack of molecular and COO data in FLYER should not affect the final conclusions of the study.

## Counterpoint

For patients with early-stage, low-risk DLBCL, 3 cycles R-CHOP followed by involved field RT is an established and widely used therapeutic option to reduce chemotherapy exposure and toxicity. Avoidance of radiation and its associated toxicities is an arguable benefit of the FLYER trial; however, it is important to accurately assess this risk in modern times. While the location of the nodal disease dictates potential toxicities, current techniques allow for refined fields of radiation and reduction in dose, minimizing short-term toxicities such as mucositis and cytopenias. Data regarding long-term toxicities, including secondary malignancy, infertility, and cardiac dysfunction, is often extrapolated from Hodgkin lymphoma (HL) given the higher utilization rates of RT. Patients with HL are typically younger at diagnosis with more life-years for development of a secondary malignancy, and often receive mediastinal radiation. This is in contrast to the older population of DLBCL (median age 66 years) with smaller radiation fields often excluding major organ systems. That being said, there are special circumstances that must be considered on an individual basis in which radiation should be avoided, such as young female patients with inguinal nodal disease to preserve fertility as well as breast and mediastinal involvement of lymphoma.

The effect of COO and high-risk molecular features in early-stage disease is inconclusive and limited to retrospective studies. Several studies as described above found no different between subtypes. An additional retrospective review of 151 patients with early-stage disease evaluated COO in patients who received R-CHOP with or without RT (13). COO was determined in 87 patients, with 62 GCB and 25 non-GCB. Excellent responses were observed in both therapeutic groups as expected. However, analysis of COO impact showed that non-GCB was associated with inferior 5-year PFS and OS (73.9% and 59.7%) compared to GCB (95.9% and 90.6%). The trend of inferior outcome was more pronounced in non-GCB receiving 4 cycles or less of R-CHOP regardless of RT. The authors of the FLYER trial plan to report biologic risk factors in their population of patients, a necessary analysis to support the safety of abbreviated therapy across all subtypes.

Long-term follow up from both the SWOG 8736 and SWOG 0014 trials shows a continued pattern of late relapses in early-stage DLBCL, suggesting that CMT and rituximab may not mitigate risk of long term relapse in this population (7, 14). This pattern was again observed in the FLYER study. While relapse rates after CR or unconfirmed CR were low at 4-5%, the majority of relapses were observed after 2 years, with the cumulative incidence of relapse demonstrating a linear trend during years 3 to 6 of follow-up. So, while the approach of abbreviated chemotherapy was demonstrated to be non-inferior to a longer course of chemotherapy, the issue of late relapse in this early-stage, favorable-risk population remains.

The extended recruitment period (11 years) should be noted and was recognized by the authors. This prolonged recruitment time is likely reflective of the low incidence of early-stage, low-risk DLBCL and of DLBCL in younger patients in general. While DLBCL is the most common type of non-Hodgkin lymphoma, only approximately 30% of patients present with stage I/II disease by PET scan (15). The median age at diagnosis is 66 years, with most patients being diagnosed between ages 65-74 years; this older population was not included in this trial (16). While the findings of this study are certainly applicable to the patient population evaluated, they are not generalizable to patients older than 60 which make for approximately 50% of all patients with early-stage DLBCL (8, 9).

## Discussion

The FLYER trial was built on the observation (from MInT) that a subset of DLBCL patients with age ≤60, zero IPI risk factors, and non-bulky disease have extraordinarily favorable outcomes, with OS close to 95% when treated with 6 cycles R-CHOP-21. Although a large fraction of these patients had early-stage DLBCL and could have been treated with CMT, they received the extended chemotherapy regimen, without radiation. This observation raised the question: can patients with very favorable DLBCL be treated with only 4 cycles R-CHOP +2 additional cycles of rituximab and still have the same excellent outcomes with less toxicity? The answer, provided by the FLYER trial, is yes. What the FLYER trial cannot tell us, because it was not designed to, is whether 4 cycles R-CHOP are equivalent to 3-4 cycles R-CHOP plus RT, in this population. As discussed above, evidence surrounding the use of RT in patients with early-stage DLBCL is complex and should be parsed carefully, to ensure the right conclusions are not applied to the wrong patients. To avoid this fallacy, reviewing the specifics of each trial is essential. Studies conducted in the pre-rituximab era (S8736 and GELA LNH-93.4) have lost much of their relevance, not only given lack of rituximab, but also because the diagnostic classification of B-cell lymphoma has changed so dramatically that one cannot be sure the same patients are evaluated in more recent studies (in fact, S8736 likely included some T-cell lymphomas). Guessing the impact of COO and molecular features on the outcomes found in these trials is also difficult, because this information was not available when the trials began. Another issue is patient selection based on definition of bulky disease, risk stratification, and age. The trials discussed above are inconsistent regarding definition of “bulk” and eligibility, and conclusions are often drawn from subset analyses of the entire cohort. Finally, emerging data on the value of iPET in adapting treatment selection *post-hoc* are gradually making *a priori* therapeutic planning based on risk stratification less relevant. Thus, NCTN S001 and LYSA/GOELAM 02-03 data provide strong support for a RT-free approach to early-stage DLBCL, for iPET-negative patients, including patients older than 60, and those with tumor bulk <10 cm. Of course, this raises the question of quality assurance in PET interpretation, which remains inconsistent. This is where the FLYER study is quite helpful, because it did not have a PET-adapted design. In conclusion, most patients with early-stage DLBCL can be treated with abbreviated R-CHOP without radiation. Patients younger than 60, with tumors <7.5 cm, and no IPI risk factors, can be treated with 4 cycles R-CHOP without iPET, based on the FLYER trial. Patients older than 60, with masses <10 cm, and some IPI risk factors, can be treated with 4 cycles R-CHOP if their iPET is negative. Long-term follow up is necessary. The studies of CMT, both before and after rituximab, suggest a pattern of late relapses, which could be due to a distinct biology. Further characterization of the biologic and clinical diversity of early-stage DLBCL is warranted.

## Author Contributions

KS and AM wrote and edited the manuscript. AB and PP reviewed and edited the manuscript. All authors contributed to the article and approved the submitted version.

## Funding

AM is supported by the National Institutes of Health institutional training grant T32GM008562.

## Conflict of Interest

The authors declare that the research was conducted in the absence of any commercial or financial relationships that could be construed as a potential conflict of interest.

The handling editor declared a shared affiliation with the authors at the time of review.
